# End-to-End Train Horn Detection for Railway Transit Safety

**DOI:** 10.3390/s22124453

**Published:** 2022-06-12

**Authors:** Van-Thuan Tran, Wei-Ho Tsai, Yury Furletov, Mikhail Gorodnichev

**Affiliations:** 1 Department of Electronic Engineering, National Taipei University of Technology, Taipei 10608, Taiwan; thuan.tranvan586@gmail.com; 2 Department of Mathematical Cybernetics and Information Technology, Moscow Technical University of Communications and Informatics, 111024 Moscow, Russia; yury.furletov@gmail.com (Y.F.); m.g.gorodnichev@mtuci.ru (M.G.); 3 Department of Automotive Engineering, Moscow Automobile and Road Construction State Technical University, 125319 Moscow, Russia

**Keywords:** audio classification, convolutional neural networks, end-to-end models, raw waveforms, railway audible warning signal, railway transit safety, train horn detection

## Abstract

The train horn sound is an active audible warning signal used for warning commuters and railway employees of the oncoming train(s), assuring a smooth operation and traffic safety, especially at barrier-free crossings. This work studies deep learning-based approaches to develop a system providing the early detection of train arrival based on the recognition of train horn sounds from the traffic soundscape. A custom dataset of train horn sounds, car horn sounds, and traffic noises is developed to conduct experiments and analysis. We propose a novel two-stream end-to-end CNN model (i.e., THD-RawNet), which combines two approaches of feature extraction from raw audio waveforms, for audio classification in train horn detection (THD). Besides a stream with a sequential one-dimensional CNN (1D-CNN) as in existing sound classification works, we propose to utilize multiple 1D-CNN branches to process raw waves in different temporal resolutions to extract an image-like representation for the 2D-CNN classification part. Our experiment results and comparative analysis have proved the effectiveness of the proposed two-stream network and the method of combining features extracted in multiple temporal resolutions. The THD-RawNet obtained better accuracies and robustness compared to those of baseline models trained on either raw audio or handcrafted features, in which at the input size of one second the network yielded an accuracy of 95.11% for testing data in normal traffic conditions and remained above a 93% accuracy for the considerable noisy condition of-10 dB SNR. The proposed THD system can be integrated into the smart railway crossing systems, private cars, and self-driving cars to improve railway transit safety.

## 1. Introduction

The railway is one of the most convenient and popular forms of public transportation that can carry a lot of people, especially during rush hours. Traveling by railway system significantly avoids traffic jams, so an accurate timetable can be achieved, which is a crucial characteristic of railway transport. To assure the smooth operation and safety of railway traffic, at the level crossings, train stations, and maintenance working zones, the passengers, pedestrians, railway service employees, and other road users should be warned of approaching train(s), so they can pay attention and cooperate appropriately. The warning signals for train arrivals can come from two sources: the signals generated by the train warning system (TWS), such as sirens, spoken warnings, and lights; and the train horns from the approaching train(s). In reality, because of the unawareness of train warning signals, which also means the unknowingness of oncoming trains, serious railway accidents sometimes happen, especially at barrier-free level crossings and track maintenance areas. Thus, the early detection and warning of train arrivals are essential for the safety and security of railway operations.

To date, different systems based on sensing techniques [1] have been applied for train arrival detection (TAD). The traditional commercialized methods include the use of treadle mechanisms, inductive sensors, and infrared beam sensors for axle-counting and determining the direction information from the approaching train. More innovative approaches have been also examined for TAD. For example, radar technology [2,3] is used near level crossings for the ranging and determination of oncoming trains, or train approach detection using the rail vibration measured by accelerometers [4]. Although the aforementioned approaches are reliable, they are not flexible because they can be only installed along with the rail structure, in which traditional methods further require significant work for installation. This work examines the TAD based on the recognition of train horn sounds from the surrounding soundscape, which is referred to as the train horn detection (THD) system or the train-horn-based TAD (TH-TAD) system. We formulate the THD as the three-class audio classification problem, where the three sound classes consist of train horn sounds, car horn sounds, and noises.

Unlike the image-related problems (e.g., image/video classification, segmentation, and object detection) where the visual input signals (i.e., images or videos) have local correlations in two spatial dimensions and two-dimensional convolutional networks (2D-CNN) are widely used to deal with the problem, the classification tasks in the audio domain involve the processing of one-dimensional signals, i.e., the audio waveforms, so different approaches have been proposed for audio classification.

In terms of deep learning-based approaches, sound classification studies come in two major groups. The first group includes the works that utilize pre-computed time–frequency representations (a.k.a. handcrafted features) of audio data as inputs and employ 2D-CNNs for classification. One of the early works in this approach is proposed in [5], in which Mel-scale spectrograms of the audio signal are extracted to feed into the 2D-CNN classifier for environmental sound classification (ESC). Using the same single-feature input, the recent works [6,7] further applied the attention mechanisms at the input layer [6,7] and/or at the output of 2D convolutional layers [7] to improve classification performance. Some follow-up studies apply the idea of the first direction to the use of other audio time-frequency representations, such as Mel-frequency cepstral coefficients (MFCCs) [8] and Gammatone-spectrogram [9], while other works [10,11] use different features in combination to train the classifiers. Recognizing that audio’s time-frequency representations are similar to single-channel images and audio classification can be regarded as the image classification task, [12] examined the use of well-known image classification models, the AlexNet [13] and GoogleNet [14], for ESC. [15] utilized more advanced techniques in the visual domain, including ResNet-50 [16] architecture, Siamese-like networks, and the attention mechanism, to achieve state-of-the-art performance in ESC. Although works in the first direction have remarkable progress in sound classification, using fixed feature extraction procedures in classification systems may result in extra processing time and the efficiency of features may depend on specific problems.

The second direction in the field of sound classification with deep learning is to directly use the audio raw wave to train one-dimensional CNN (1D-CNN) classifiers and eliminate the processes of data pre-processing and fixed feature extraction from classification systems. The advantage of this direction is that it allows for building models that perform the internal transformation from original signals to useful discriminative features that can maximize performances on specific tasks. [17] proposed 1D-CNN models for ESC and examined the effects of different factors such as input sizes and layer initialization using the Gammatone filterbank. [17]’s proposed approaches outperformed the baseline models based on 2D handcrafted inputs, showing the potential of the second direction. [18] introduced another 1D-CNN-based model, namely the SoundNet, to learn deep natural sound representation from a large amount of unlabeled data, which brings about significant performance improvements compared to the results on standard benchmarks for acoustic scene classification. Instead of using fully 1D-CNN for representation transformation, Envnet in [19] employed 1D-CNN and 2D-CNN together, in which the 1D-CNN part learned spectrogram-like inputs for the classification part formed by 2D-CNN. It is worth mentioning that the existing works [17,18,19] based on raw wave inputs reported better or comparable accuracies compared to those of 2D-CNNs with pre-computed spectrograms.

Inspired by the promising results of prior works based on raw wave inputs, our work applies this direction to build an end-to-end classifier for the train horn detection task. In addition, other techniques, such as regularization and audio data augmentation, are utilized to alleviate the overfitting problem as well as to improve the model’s generality. Data augmentation is a useful technique in training deep networks, especially in case of data scarcity as this technique helps to increase the diversity of training data, thus, efficiently preventing overfitting. It has been shown in existing works [9,20,21,22] that using data augmentation can bring about significant performance improvements in sound classification. The augmentation of audio data can be conducted either on the raw waveform or on the time-frequency representation, for instance [21] applied time-stretching, noise-adding, and pitch-shifting to original wave signals, while [22,23] performed augmentation transformations on the spectrograms.

The main contributions of this work can be summarized as follows. We introduce a modern TAD system based on train horn detection using deep learning approaches. The advantage of train-horn-based TAD is that it can be flexibly applied to mobile objects such as road vehicles, track maintenance vehicles, and smart devices that require the active detection and warning of train arrivals. Apart from collecting a custom experimental dataset (i.e., THD dataset), we introduce a novel two-stream end-to-end CNN-based audio classifier, namely THD-RawNet, for THD, in which raw audio waveforms are directly employed to build the model rather than using pre-computed features such as the widely used Mel-scale spectrogram, gammatone-based spectrograms, and Mel-frequency cepstral coefficients (MFCCs). The novelty of the proposed method is as follows. The THD-RawNet processes raw inputs in two directions simultaneously, then combines the outputs to perform final predictions. First, we propose to transform audio raw waveforms into a 3D image-like representation using three sets of 1D convolutional (1D-Conv) layers with different filter sizes, then utilize 2D-CNN to classify the 3D representation, which is partially similar to the image classification problem. Second, high-level features of raw waves are extracted using a series of 1D-Conv layers and pooling layers. The existing works only considered a single temporal resolution of the audio signal in the feature extraction process or only examined the combined use of raw input and precomputed features. Furthermore, this work proposes to conduct data augmentation with raw waveforms from all channels of stereo audio. In comparison with the performances of baseline models on the THD dataset, THD-RawNet is much more performant. The proposed TH-TAD solution based on THD-RawNet can be potentially applied to real-world applications, for instance, to improve the safety function in road vehicles and for smart monitoring at level crossings.

## 2. Materials and Methods

**Figure 1:**
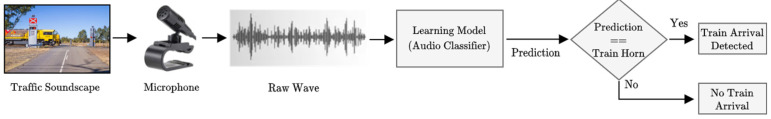
The general structure of the TH-TAD system.

For effective evaluation, we roughly assume that an audio signal from the traffic soundscape can belong to one of three classes, including train horn sounds, car horns generated by ordinary cars, and noises. The consideration of car horn and noise classes is useful to evaluate how well the proposed system can distinguish train horn sounds from similar vehicle warning sounds (i.e., car horns) and background noises in the downtown street environment. Figure 1 shows the overall structure of the train-horn-based TAD (TH-TAD) system that contains an audio recorder for continuously capturing audio data from the surrounding soundscape and an audio classifier for predicting class probabilities for every audio segment, from which the system can determine the status of train detection. The detection of the train is confirmed if the input signal is hypothesized as the train horn sound, which means that the audio classifier outputs the highest probability for the train horn class. We aim to develop a complete end-to-end audio classifier, so the objective model works directly with the audio raw wave rather than pre-computed features.

### 2.1. The Proposed THD-RawNet

**Figure 2:**
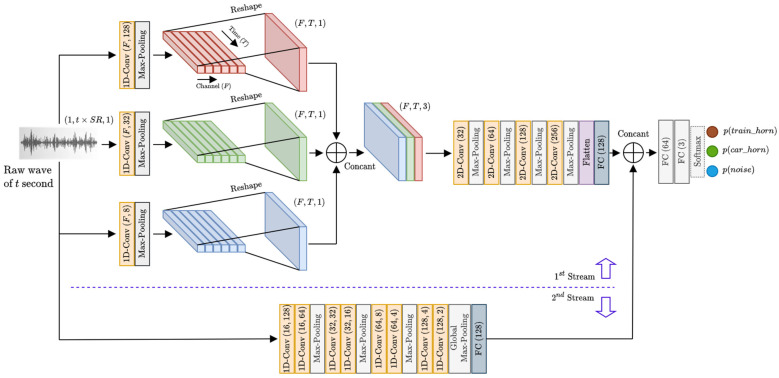
The general structure of the THD-RawNet in the TH-TAD system. SR is the sampling rate, t is the input length (in seconds), F is the number of filters in a 1D-Conv layer, “Concat” stands for concatenation operation, and FC denotes a fully connected layer.

The proposed end-to-end model, namely THD-RawNet, is illustrated in Figure 2. Given an audio segment or recording of t seconds and sampled at the sampling rate SR, the input of THD-RawNet has the shape of (1,t×SR,1) corresponding to the channel-last dimension ordering, where t×SR is the number of data points of the audio segment. As illustrated in Figure 2, the THD-RawNet consists of two streams, the upper stream utilizes both 1D and 2D convolutional layers while the bottom one is fully composed of 1D convolutional layers. Unlike the bottom stream which focuses only on extracting features along the time dimension, the upper stream converts the 1D wave signal into an image-like representation and utilizes visual-based 2D-CNN to further process the converted representation. The high-level features extracted by the two network streams are combined and fed into the remaining fully connected layers for prediction. This concept for the two-stream structure of THD-RawNet is inspired by an assumption that applying two different feature processing approaches together can help to extract more useful discriminative features, resulting in better performances. The details of each stream’s structure are as follows.

To automatically transform raw data into an image-like representation formed by different channels of 2D feature maps, the first stream of THD-RawNet is designed with three branches of 1D convolutional (1D-Conv) layers, in which the 1D-Conv layers in each branch have a distinctive filter size to learn feature representation of a specific temporal resolution. Specifically, three sets of 1D-Conv layers in three branches have large filter sizes (i.e., 128), medium filter sizes (i.e., 32), and small filter sizes (i.e., 8), respectively. The 1D-Conv layer with large filter sizes is useful to learn low-frequency features, while higher-frequency features are extracted by the 1D-Conv layers with smaller filter sizes. Each 1D-Conv layer is followed by a 1D max-pooling layer which plays the role of dimensional reduction. Note that the output at the 1D max-pooling layer has a shape of (1,T,F) where F denotes the number of filters in the 1D-Conv layer, resulting in an output tensor of F channels, and T is the number of elements in the time dimension. It is assumed that each channel of the 1D max-pooling layer’s output represents coefficients for a frequency band along the time dimension. Thus, we reshape the (1,T,F) output to (F,T,1) representation, which is similar to a time-frequency spectrogram or a single-channel image. Concatenating the outputs of three branches along the channel dimension, we obtain an image-like representation of shape (F,T,3), which is fed into the 2D-CNN structure for further processing.

In our experiments, we set the number of 1D-Conv’s filters in each branch to 128, and the corresponding 1D max-pooling layer to have the kernel size of 346 and stride of 173, which is equivalent to a sliding window of approximately 16 ms with 50% overlapping (i.e., 8 ms) at the sampling rate of 22.05 kHz. Thus, for the input segments of 1 s, we obtain the image-like representation of shape (128,22,050/173,3) or (128,128,3) corresponding to (frequency, time, channel) format, where * is the ceiling function. In the 2D-CNN part of the first network stream, the first 2D-Conv layer is configured with 32 filters, a large receptive field of (5,5), and a stride of (1,1) to take the general view of the input features. The second 2D-Conv layer with 64 filters is responsible for learning patterns along the frequency dimension (vertical dimension), and this layer has a receptive field of (3,1) and stride of (1,1). Similarly, the third 2D-Conv layer of 128 filters, receptive field (3,1), and stride (1,1) is used to learn patterns along the time dimension (horizontal dimension). The last 2D-Conv layer has 256 filters with (3,3) receptive fields to learn features in time and frequency dimensions jointly. Note that all four 2D-Conv layers are followed by 2D max-pooling layers with the stride of (1,1) to reduce the dimensions of feature maps. The last layer of the first stream is a fully connected layer of 128 neurons, whose input is the flattened vector of the last 2D max-pooling layer’s output.

As for the second stream of TH-RawNet, a chain of the stacked 1D-Conv layers and 1D max-pooling layers are utilized for feature extraction. There are four pairs of 1D-Conv layers in this stream, in which the number of filters in the ${\left(i+1\right)}^{th}$ pair double that of the ${\left(i\right)}^{th}$ pair, the layers in ${\left(i+1\right)}^{th}$ pair have smaller filter sizes compared to those of layers in the ${\left(i\right)}^{th}$ layer, and the first three pairs of 1D-Conv layers are followed by a max-pooling layer to reduce the size of output along the time dimension. Among eight 1D-Conv layers, the first two layers with large filter sizes (i.e., 128 and 64) play the role of catching the global view of the raw wave signal and extracting the local features, while the other layers are responsible for getting a more in-depth view of the data to find more useful discriminative features for the classification task. We downsample the output of the last 1D-Conv layer by taking the maximum value over the time dimension, thus obtaining the input for the fully connected layer of 128 neurons. We can see that both streams of THD-RawNet end with fully connected layers. Therefore, we can simply concatenate the outputs of two streams to form the combined feature vector that is fed into the other fully connected layers for classification. The final layer has three neurons with the softmax activation function to generate three class probabilities p(c) for an input, where c∈classes={train_horn, car_horn, noise}, from which the system can determine the status of train horn detection based on the result of decision rule (1). If the THD-RawNet outputs the highest probability on train horn class (i.e., $c^{*}=train\_horn$) the train horn is detected. On the other hand, if $c^{*}\neq train\_horn$, the audio segment is hypothesized as a car horn sound or noise, so no train horn is detected.

(1) $$c^{*}=\underset{c\in classes}{\arg\;\max}p\left(c\right)$$

### 2.2. Data Collection

There is no published data for TH-TAD, so we create a custom dataset of three sound classes, including train horn sounds, car horn sounds, and noises. We utilize different approaches to collect real-field recordings that are captured near railway systems and other places of urban traffic. Firstly, we extract relevant recordings from online resources specialized in audio/video clips of train arrivals and traffic soundscape. By accessing the YouTube video-sharing framework we find and extract a large number of videos about train arrival recorded all over the world, which provides a diverse database of train horn sounds, and railway noises. Secondly, we extract more data from the relevant published dataset, the ESC-50 dataset [24], which provides car horn sounds and various types of urban noises. Lastly, we record real audio clips of Taiwan’s railway and urban traffic.

The recordings from online sources and real-field recordings are split into non-overlapping clips of 2 s, resulting in 5289 train horn samples, 5848 car horn samples, and 4302 noise samples. We then combine the collected data with that of ESC-50, which contains 40 car horn samples and 1960 noise samples, to form the complete dataset of 17,399 samples, as shown in Table 1. Note that ESC-50 includes various sets of noises such as exterior/urban noises, interior/domestic sounds, natural soundscapes, and human-non-speech sounds, so ESC-50 complements our collected data to create a relatively diverse sizeable dataset. The whole data are organized in three subsets (i.e., training set, validation set, and testing set) following the rule that the original recordings in a subset are different from those of the other subsets. In each subset, there is an approximately equal amount of audio samples for each sound class. The detail of data separation is shown in Table 2.

### 2.3. Waveform-Based Data Augmentation

**Figure 3:**
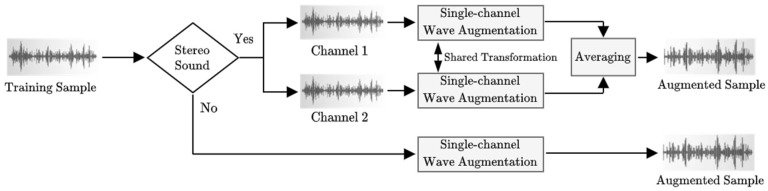
The procedure for the augmentation of training data.

It is not always easy to collect a larger amount of data with good variability to train neural networks, and this work is not an exception. Thus, data augmentation (DA) is used to artificially generate additional training data, thereby improving the system performance, mitigating overfitting, and enhancing the system generality. Since the proposed THD-RawNet works with raw input, we only conduct waveform augmentations with four transformations, including background noise addition, time-cyclic, time-stretching, and random-gain. Our TH-TAD data cover both mono and stereo samples, so we employ different augmentation procedures for those two types of inputs, as presented in Figure 3. A mono or single-channel sample is processed directly with one of four candidate transformations to generate the augmented sample, which is referred to as single-channel wave augmentation (SCWA). For stereo samples, SCWA is separately performed on each channel of the training sample, then the results are averaged to create the final augmented single-channel sample. To assure temporal alignment and avoid abnormal combinations, two channels of a stereo sound share the same transformation with random parameters. The size and label of the augmented sample are the same as those of the original training sample.

In the noise addition approach, we mix an original sample with a noise sample using (2), which yields a noisy augmented signal a, where o is the original signal, n is the noise, and w∈(0, 1) is a random weight. To perform time-cyclic augmentation, we randomly shift an original signal by a random number of data points (i.e., by 30% to 70% of the signal size), so the signal is separated into two parts, then the second part is placed in front of the first part to create the augmented sample. In time-stretching, we change the speed of the audio sample according to a random rate. Lastly, random-gain augmentation is used to scale the amplitude of an original signal by a random ratio.

(2) a=(1−w)·o+w·n

## 3. Results and Discussion

### 3.1. Experiment Setup

The experimental data were collected from different sources and contained various sampling rates, so all audio samples were normalized with the sampling rate of 22.05 kHz, which was performed using Librosa [25], a useful python library for audio signal processing. Although almost all recordings in the experimental dataset are between 2s and 5s, we only examine the input length of 1s since using a shorter input can reduce the computational complexity of the models, especially for the end-to-end models trained on raw wave signals. Short input is also favorable for the practical TH-TAD application, which requires a relatively quick response and continuous prediction. We process long samples to train networks with a fixed input length of one second as follows. The audio sample is split into non-overlapping segments of one second, and those segments share the same label as the original sample. Performing this process on training data results in a larger number of data samples and thus can be viewed as another sort of data augmentation. For the testing phase, the classification prediction (i.e., $p{\left(c\right)}_{X}$) for a long testing sample X is obtained by aggregating the predictions of all one-second segments using the sum rule, which is presented by (3), where $p{\left(c\right)}_{i}$ is the network’s prediction for the $i^{th}\;\left(i=1,\ldots ,\;S\right)$ segment of sample X. S is the total number of segments and c∈classes={train_horn, car_horn, noise}. We make the final decision based on the maximum $p{\left(c\right)}_{X}$ value, as presented in (4).

(3) $$p{\left(c\right)}_{X}=\frac{1}{S}\overset{S}{\underset{i=1}{\sum }}p{\left(c\right)}_{i}$$

(4) $$c^{*}=\underset{c\in classes}{\arg\;\max}p{\left(c\right)}_{X}$$

The basic setup to train deep learning models in our experiments is as follows. The categorical cross-entropy acts as the loss function. Models are trained using the Adam optimizer [26] with an initial learning rate of 0.00001. We additionally utilize batch normalization [27] for all layers to speed up the training process, and dropout regularization [28] is applied to alleviate overfitting. We set the batch size to 16, and training data is shuffled after every training epoch. To analyze the robustness of the proposed model and the baseline models, we report their performances on noisy testing sets of different signal-to-noise (SNR) levels consisting of +15 dB, +10 dB, +5 dB, 0 dB, −5 dB, −10 dB, and −15 dB. To create testing sets with the aforementioned SNRs, we conduct the artificial addition of noises to the original testing set, in which weather sounds, including strong wind sounds and rain sounds, are utilized as the noise sources. It is worth mentioning that the original testing samples are collected in the real traffic soundscape, so they already contain background noises at certain levels. Therefore, noisy testing sets generated by artificial noise addition create more challenging evaluation conditions for the proposed models. The noise recordings used for training data augmentation are different from those for the creation of noisy testing data. In addition, the SNRs in noisy testing sets are almost unseen by the models because training data augmentation was performed randomly without specifying any SNR ratios for noise addition.

### 3.2. Performance of Proposed THD-RawNet

Table 3 shows the performances of the proposed end-to-end THD-RawNet and several baseline models on the THD dataset. To make a comparative analysis, apart from models based on raw wave input (i.e., SoundNet [18] and EnvNet [9]), we also considered those trained with precomputed time-frequency input (i.e., Mel-scale spectrogram), including 2D-CNN [5,13,21], a recurrent neural network (RNN [29]), and a convolutional recurrent neural network (CRNN [7]). From Table 3 we can see that the proposed THD-RawNet provides a much better accuracy (95.11%) compared to those of baseline models trained with either raw inputs or handcrafted features. For the case of raw wave inputs, variants of SoundNet [18], the deep networks composed of five or eight stacked 1D-Conv layers, yielded the accuracies of 90.17%, and 92.17%, which are 4.94% and 2.94% lower than the results of the proposed THD-RawNet, respectively. Similarly, the EnvNet [9], which combines the use of 1D-CNN and 2D-CNN, also produced a moderate accuracy (88.23%). For the approach based on 2D-CNN, RNN, and CRNN with Mel-scale spectrogram input, the accuracies are almost the same across the five existing models we examined, in which one of the deepest models, the AlexNet [13], yielded the highest accuracy (90.05%) among the five models, but this figure is 5.06% lower than that of THD-RawNet. In terms of computational complexity, the THD-RawNet requires more processing time for a single prediction with a 1-s audio signal, at 5 ms, which is slightly larger than the time ranging from 1 ms to 3 ms of SoundNet [18], EnvNet [9], CRNN [7], and three 2D-CNN models in [5,13,21]. However, the computational time of the proposed THD-RawNet is much smaller than that of the RNN model [29]. More importantly, the inference time of 5 ms per sample is fairly small and can meet the real-time processing requirement in practical applications.

**Figure 4:**
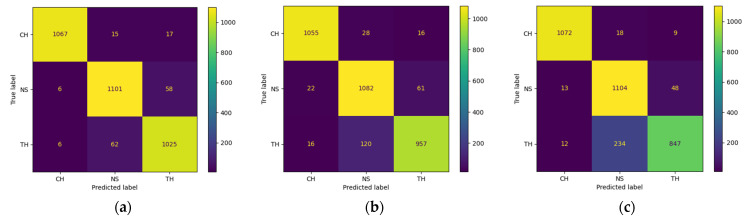
Confusion matrices for THD-RawNet, SoundNet (8 Conv layers), and AlexNet. CH, NS, and TH denote the car horn, noise, and train horn classes, respectively. (**a**) THD-RawNet. (**b**) SoundNet (8 Conv layers). (**c**) AlexNet.

Figure 4 provides confusion matrices associated with predictions of THD-RawNet, and two baseline models, the SoundNet (eight 1D-Conv layers) and the AlexNet, which received the highest accuracies for raw input and spectrogram input, respectively. We can see that the major misclassification rates in all three models are between noise class (NS) and train horn class (TH). Each model misclassified the equivalent number of noise samples to train horn sounds (i.e., THD-RawNet (58), SoundNet (61), and AlexNet (48)). However, the misclassification rates due to classifying train horn sounds into noise classes are much different across the three models. Specifically, THD-RawNet incorrectly classified 62 train horn samples into noises, while the figures for SoundNet and AlexNet are 120 and 234 samples, which are approximately two times and four times larger than the figure for THD-RawNet, respectively. Thus, THD-RawNet is more efficient at increasing the correct predictions for train horn samples. For the car horn and noise classes, the three models achieved almost the same performance.

### 3.3. Effects of Multiple Temporal Resolution Approach and Two-Stream Architecture

In this experiment, we evaluated the effectiveness of the proposal to process raw data simultaneously with multiple temporal resolutions in the first stream of THD-RawNet, where 1D-CNNs transform raw waves into 2D feature maps to feed into the 2D-CNN part. We examined the performance of the first stream configured with one, two, or three 1D-CNN branches, in which the 1D-Conv layer in each branch has 128 filters and can be set with a large filter size (128), a medium filter size (32), and a small filter size (8). We do not consider the configurations for 1D-Conv layers with filer sizes larger than 128 because those configurations result in high computational costs. Table 4 provides the results of this experiment. Considering the case that the first stream of THD-RawNet has a single branch of 1D-CNN, we obtained the accuracies of 92.25%, 89.66%, and 87.85% for the cases of large (128), medium (32), and small (8) filter sizes, respectively. We can see that the larger the filter size the better accuracy we achieve, and it is assumed that the larger filter size allows the 1D-Conv layer to observe longer dependencies in the raw inputs, from which the layer can extract useful features for the classification task. The combined use of multiple 1D-CNN branches to build the first stream of THD-RawNet also brings about better performances, in which combining a branch with a large filter size with either a medium filter size branch or a small filter size branch results in a significant accuracy improvement, to 92.85% and 92.40%, respectively. Similarly, a two-branch structure with a medium filter size branch and a small filter size branch achieves 90.65% accuracy, which is 1% and 2.8% higher than the results of two corresponding single-branch structures, respectively. Lastly, the proposed three-branch structure, as illustrated in the upper stream of Figure 2, obtained the highest accuracy (93.68%) among all examined structures for THD-RawNet’s first stream, further showing that utilizing multiple temporal resolutions together can significantly improve the classification accuracy in THD.

Next, we analyzed the effectiveness of the proposed two-stream architecture of THD-RawNet, in which we performed separate experiments on the first stream and second stream of the network and compared the results with that of the two-stream structure. As shown in Table 5, the first stream based on multiple 1D-CNN branches and 2D-CNN classification yielded an accuracy of 93.68%, while the second stream built with fully 1D-Conv layers produced an accuracy of 92.52%. It is worth mentioning that both streams achieved much better performances compared to those of baseline models [5,7,9,13,18,21,29], showing the efficiency of our proposed architectures for two network streams of THD-RawNet. By combining two streams to form the proposed THD-RawNet, we achieved a considerable improvement in classification accuracy, to 95.11%, which is 1.43% and 2.59% higher than the results of the first stream and the second stream, respectively. It is noted that combining two network streams to form the THD-RawNet results in a small increase in computational time. Specifically, the processing time of two-stream THD-RawNet for a 1-s audio segment is 5 ms, which is higher than the figures for each stream of THD-RawNet by 1 ms and 3 ms, respectively.

### 3.4. Robustness Evaluation

In this experiment, we evaluated the robustness of the proposed THD-RawNet and made a comparison with that of the baseline models. We tested the pre-trained models with testing sets of various noise levels, including −15 dB, −10 dB, −5 dB, 0 dB, +5 dB, +10 dB, and +15 dB. From the statistic in Table 6, it is shown that the THD-RawNet has the best performance. Across all noise levels, the THD-RawNet yielded much higher accuracies compared to those of the baseline models trained with either raw wave or precomputed features. At the moderate noisy conditions, i.e., the SNRs of +15 dB, +10 dB, and +5 dB, the performances of all models reduced slightly to obtain almost comparable accuracies as in the case of original testing data (or relatively clean testing set). For the noise level of 0dB, the accuracy of THD-RawNet decreased by less than 1%, while the accuracies of the baseline models started reducing more significantly, by 2% to 4%. In more challenging conditions, i.e., SNRs of −5 dB and −10 dB, a greater difference in performances of the THD-RawNet and the baseline models was observed, in which the THD-RawNet still attained high accuracies, at 93.74% (−5 dB) and 93.08 (−10 dB). By contrast, the baseline models experienced huge performance degradation, by at least 3.81% (CRNN [7]) for −5 dB testing data, and by approximately 4.5% (2D-CNN (K. J. Piczak [5]) to 11% (EnvNet [9]) for SNR of −10 dB. As for the noisiest condition (−15 dB), the accuracies of all the models reduced dramatically, but the figure for the THD-RawNet remained above 82.90%, while the accuracies of the baseline models were smaller than 77.86%.

We also conducted the same experiments with each stream of the THD-RawNet and inferred three observations. First, both network streams attained better robustness than the existing models, especially for the cases of negative SNRs where the baseline models’ performances degraded significantly while each of the THD-RawNet’s streams still yielded high accuracies. For example, at an SNR of −10 dB, the accuracies of the first stream and second stream are 89.51% and 88.35%, respectively, whereas the figures for the baseline models are 5% to 30% smaller. Second, considering the first stream with the recall that this stream and EnvNet [9] apply and the similar idea of converting raw waves to time–frequency-like features with 1D-CNN and classification with 2D-CNN, we can see that the use of multiple 1D-CNN branches in the first stream of the THD-RawNet resulted in better robustness. Specifically, the performance of EnvNet, the network with a single 1D-CNN branch, degraded much more significantly than the first stream of THD-RawNet, the model with three 1D-CNN branches. Third, in all levels of noise, by combining two network streams the resulting THD-RawNet obtained better accuracies compared to those of each stream and mitigated accuracy reduction when the noise level was increased. This further proves the effectiveness of the proposed two-stream structure for the THD-RawNet.

### 3.5. THD-RawNet Performances with Different Input Sizes

Table 7 shows the performances of the proposed THD-RawNet for the other input lengths rather than one second, including 0.25 s, 0.5 s, 0.75 s, and 2 s. Generally, the shorter the input length the lower the accuracy yielded by the model is, and the processing time is smaller. Training the model with data of 2 s, we achieved an accuracy of 95.53%, which is 0.42% higher than that of the model trained on 1-s input. However, the processing time per 2-s sample (10 ms) is double that for a 1-s sample (5 ms). For an input size of 0.75s, THD-RawNet produced almost a comparable accuracy (94.81%) in the case of 1-s data (95.11%). When it comes to 0.5 s and 0.25 s inputs, the accuracy of THD-RawNet decreased more significantly, but remained above 90%, at 93.71% and 92.11% for 0.5 s and 0.25 s inputs, respectively. Although the inference times are much different between long input sizes (i.e., 2 s) and short input sizes (i.e., 0.5 s and 0.25 s), inference times in all cases are short enough and well acceptable for practical application. Among five cases of input lengths, 0.75 s and 1 s could be most suitable for the THD task using the THD-RawNet.

## 4. Conclusions

This work studied the end-to-end deep learning-based approach for train horn detection (THD), which is applied for train arrival detection (TAD) in rail transit safety. The task was regarded as an audio classification problem of three sound classes consisting of train horn sounds, car horn sounds, and background noises. We proposed a novel two-stream end-to-end convolutional neural network, the THD-RawNet, to utilize as the audio classifier of the THD system, in which the network worked directly with raw audio waveforms rather than precomputed features such as a spectrogram and MFCCs. The THD-RawNet is composed of two network streams to combine two approaches for processing raw audio waveforms with 1D-Conv layers, one stream is a sequential 1D-CNN model with 1D-Conv layers, 1D max-pooling layers, and fully connected layers, while in the other stream, we propose to convert raw waves into an image-like representation using multiple branches of 1D-CNN considering different temporal resolutions.

Conducting experiments on the custom dataset, we found that the THD-RawNet outperformed various baseline models trained with either raw waves or precomputed features (i.e., Mel-spectrogram). THD-RawNet attained a good level of robustness as its accuracies dropped modestly in experimental noisy conditions with SNRs ranging from −10 dB to +15 dB. Our experiments also showed the effectiveness of the two-stream structure in the THD-RawNet as well as of the multiple temporal resolution approach utilized in a stream of the THD-RawNet. By considering different temporal resolutions in the conversion of raw waveforms to time–frequency-like representation, we achieved a considerable improvement in classification accuracy. Equally important, the higher accuracy of two-stream THD-RawNet compared to the performances of each network stream has shown the complementary relationship of features extracted from raw waveforms, respectively, by 1D-CNN and 1D2D-CNN architectures. In addition, an investigation regarding the performance of the THD-RawNet with respect to different input lengths was conducted, showing that 0.75 s or 1s are reasonable input sizes that can balance the accuracy and speed requirements of practical applications. In comparison with baseline approaches, the proposed method attained much better accuracies with a slight increase in computational time.

Although we have achieved promising preliminary results, limitations do exist, and further efforts are required to enhance the applicability of the proposed methods. At the current stage of our research, the experimental dataset may not perfectly reflect the real traffic soundscape yet, and the determination of train horn direction has not been investigated. In future work, we would further consider some vital aspects of the train horn detection problem as follows. In terms of the experimental dataset, more data would be collected with the consideration of training data diversity, covering more complex traffic scenarios and weather conditions. Especially, we will examine the scenario where several sounds may be presented at the same time. Besides, techniques for noise removal and sophisticated data augmentation would be applied to improve the robustness of the audio classifier. The combination of raw waves and handcrafted features could be also taken into consideration to examine the complementary relationship between those two feature sets. Furthermore, we would examine the problem of direction determination for train horn sounds, which is another essential aspect of the THD applications.

## Figures and Tables

**Table 1:** The summary of our data preparation.

Data Class	Data Sources		Total (#Samples)
	Our Collection	ESC-50	
Train Horn	5289	-	5289
Car Horn	5808	40	5848
Noise	4302	1960	6262
Total (#samples)	15,399	2000	17,399
Total duration	8.55 h	2.77 h	11.32 h
Clip length	2 s	5 s	-

**Table 2:** Data separation for TH-TAD experiments.

Subset	Train Horn	Car Horn	Noise	Total
Train	3211	3624	3871	10,706
Validation	985	1225	1226	3336
Test	1093	1099	1165	3357
Total	5289	5848	6262	17,399

**Table 3:** Performance of the proposed THD-RawNet and baseline models on the THD dataset.

Model	Input/Features	Inference Time (ms/Sample)	Accuracy (%)
THD-RawNet (this work)	Raw wave	5 ms	95.11
SoundNet (5 Conv layers [18])	Raw wave	1 ms	90.17
SoundNet (8 Conv layers [18])	Raw wave	2 ms	92.17
EnvNet [9]	Raw wave	2 ms	88.23
2D-CNN (K. J. Piczak [5])	Mel-scale spectrogram	3 ms	89.04
2D-CNN (J. Salamon et al. [21])	Mel-scale spectrogram	1 ms	89.90
2D-CNN (AlexNet [13])	Mel-scale spectrogram	3 ms	90.05
RNN (I. Lezhenin et al. [29])	Mel-scale spectrogram	8 ms	80.22
CRNN [7]	Mel-scale spectrogram	2 ms	87.99

**Table 4:** Performance of the first stream of THD-RawNet with different configurations.

Model	#Branches	1D Filter Size in Each Branch	Output of 1D-CNN part	Accuracy (%)
1st Stream of THD-RawNet	1	Large (128)	(128,128,1)	92.25
1st Stream of THD-RawNet	1	Medium (32)	(128,128,1)	89.66
1st Stream of THD-RawNet	1	Small (8)	(128,128,1)	87.85
1st Stream of THD-RawNet	2	Large (128), Medium (32)	(128,128,2)	92.40
1st Stream of THD-RawNet	2	Large (128), Small (8)	(128,128,2)	92.85
1st Stream of THD-RawNet	2	Medium (32), Small (8)	(128,128,2)	90.65
1st Stream of THD-RawNet	3	Large (128), Medium (32), Small (8)	(128,128,3)	93.68

**Table 5:** Performances of the proposed THD-RawNet and its two streams.

Model	Features	Inference Time (ms/Sample)	Accuracy (%)
THD-RawNet	Raw wave	5 ms	95.11
1st Stream of THD-RawNet	Raw wave	4 ms	93.68
2nd Stream of THD-RawNet	Raw wave	2 ms	92.52

**Table 6:** Results of proposed THD-RawNet and baseline models across various levels of noise.

Models	Input/Features	Accuracy (%) on Each SNR							
		−15 dB	−10 dB	−5 dB	0 dB	+5 dB	+10 dB	+15 dB	Original Data
THD-RawNet (this work)	Raw wave	82.90	93.08	93.74	94.31	94.51	94.66	94.70	95.11
1st Stream of THD-RawNet (this work)	Raw wave	80.16	89.51	92.25	92.52	92.76	93.39	93.68	93.68
2nd Stream of THD-RawNet (this work)	Raw wave	79.56	88.35	91.45	91.71	92.01	92.04	92.37	92.52
SoundNet (five Conv layers [18])	Raw wave	71.02	80.87	86.53	88.44	89.18	89.24	89.78	90.17
SoundNet (eight Conv layers [18])	Raw wave	75.93	84.27	88.53	90.11	90.49	90.55	91.39	92.17
EnvNet [9]	Raw wave	72.08	77.06	83.37	85.25	85.79	86.71	87.01	88.23
2D-CNN (K. J. Piczak [5])	Spectrogram	77.45	84.59	85.23	86.92	87.42	88.47	88.62	89.04
2D-CNN (J. Salamon et al. [21])	Spectrogram	77.86	84.62	85.43	86.62	88.17	88.88	89.87	89.90
2D-CNN (AlexNet [13])	Spectrogram	77.21	83.26	85.79	86.38	87.60	88.44	89.06	90.05
RNN (I. Lezhenin et al. [29])	Spectrogram	55.46	58.26	65.65	70.74	72.00	75.96	78.37	80.22
CRNN [7]	Spectrogram	75.66	81.14	84.18	84.77	85.56	86.77	87.75	87.99

**Table 7:** Performances of THD-RawNet with different input sizes.

Input Size (s)	0.25 s	0.5 s	0.75 s	1 s	2 s
Accuracy	92.11%	93.71%	94.81%	95.11%	95.53%
Inference time (ms/sample)	1 ms	2 ms	4 ms	5 ms	10 ms

## Data Availability

The data that support the findings of this study are available from the authors upon reasonable request.
